# Role of the SLC26A9 Chloride Channel as Disease Modifier and Potential Therapeutic Target in Cystic Fibrosis

**DOI:** 10.3389/fphar.2018.01112

**Published:** 2018-10-01

**Authors:** Anita Balázs, Marcus A. Mall

**Affiliations:** ^1^Department of Pediatric Pulmonology, Immunology and Intensive Care Medicine, Charité – Universitätsmedizin Berlin, Berlin, Germany; ^2^Berlin Institute of Health, Berlin, Germany; ^3^German Center for Lung Research, Giessen, Germany

**Keywords:** cystic fibrosis, epithelial ion transport, chloride channels, SLC26A9, pharmacology

## Abstract

The solute carrier family 26, member 9 (SLC26A9) is an epithelial chloride channel that is expressed in several organs affected in patients with cystic fibrosis (CF) including the lungs, the pancreas, and the intestine. Emerging evidence suggests SLC26A9 as a modulator of wild-type and mutant CFTR function, and as a potential alternative target to circumvent the basic ion transport defect caused by deficient CFTR-mediated chloride transport in CF. In this review, we summarize *in vitro* studies that revealed multifaceted molecular and functional interactions between SLC26A9 and CFTR that may be implicated in normal transepithelial chloride secretion in health, as well as impaired chloride/fluid transport in CF. Further, we focus on recent genetic association studies and investigations utilizing genetically modified mouse models that identified SLC26A9 as a disease modifier and supported an important role of this alternative chloride channel in the pathophysiology of several organ manifestations in CF, as well as other chronic lung diseases such as asthma and non-CF bronchiectasis. Collectively, these findings and the overlapping endogenous expression with CFTR suggest SLC26A9 an attractive novel therapeutic target that may be exploited to restore epithelial chloride secretion in patients with CF irrespective of their *CFTR* genotype. In addition, pharmacological activation of SLC26A9 may help to augment the effect of CFTR modulator therapies in patients with CF carrying responsive mutations such as the most common disease-causing mutation F508del-CFTR. However, future research and development including the identification of compounds that activate SLC26A9-mediated chloride transport are needed to explore this alternative chloride channel as a therapeutic target in CF and potentially other muco-obstructive lung diseases.

## Introduction

Cystic fibrosis (CF) is a severe life-shortening multiorgan disease caused by mutations in the gene encoding the CF transmembrane conductance regulator (CFTR) chloride channel, which plays a fundamental role in salt and fluid transport across the surfaces and ducts of many epithelial organs including the lungs, pancreas, and gastro-intestinal tract. In patients with CF, CFTR dysfunction results in impaired epithelial ion and water transport that leads to a characteristic multi-organ pathology (Mall and Hartl, 2014). More, than 2000 *CFTR* variants have been identified in CF patients, with F508del being the most common accounting for 70% of all CF mutations (Sosnay et al., 2013). Disease-causing mutations may lead to various molecular defects in CFTR protein production, processing, channel function, and stability at the plasma membrane (Welsh and Smith, 1993; Mall and Hartl, 2014). Drug development efforts aiming to restore CFTR function have yielded mutation specific pharmacotherapies that are now available for a subgroup of patients with CF (Gentzsch and Mall, 2018). These include the CFTR potentiator compound ivacaftor (VX-770) that can improve channel activity in CFTR gating mutations present in ∼5% of CF patients and the combination of ivacaftor with the CFTR corrector compound lumacaftor (VX-809) that has been approved for F508del homozygous patients (Ratjen et al., 2017). The large number of disease-causing variants and the variability of biological effects of CFTR mutations and their responsiveness to CFTR modulator therapies argues that targeting an alternative chloride channel may be an attractive therapeutic strategy in CF (Li et al., 2017). Emerging evidence suggests that the alternative chloride channel SLC26A9 is a modulator of CFTR function and a potential candidate to circumvent the primary ion transport defect in several organs affected by CF including the lungs, the pancreas, and the gastro-intestinal tract independent of the *CFTR* genotype (Li et al., 2017; Gentzsch and Mall, 2018). In this review, we summarize the basic physiological and pharmacological properties of SLC26A9, outline its regulation and molecular interaction with CFTR, detail the role of SLC26A9 as a disease modifier in CF and potentially in other diseases, and finally discuss the impact of these discoveries on future research that is needed to explore this alternative chloride channel as a potential therapeutic target in the clinical arena.

## Physiology, Pharmacology, and Regulation of SLC26A9

SLC26A9 is a member of the solute-linked carrier 26 (SLC26) anion transporter family that functions uniquely as a chloride channel with minimal conductance to bicarbonate (Dorwart et al., 2007; Loriol et al., 2008). SLC26A9 is mainly expressed in epithelial cells of the respiratory tract, stomach, duodenum, ileum, and the pancreas, while transcripts were also detected in the salivary gland, kidney, brain, heart, prostate, testis, ovary, and skin (Lohi et al., 2002; Xu et al., 2005; Chang et al., 2009b; Lee et al., 2015; Liu et al., 2015; Consortium, 2017). SLC26 members share a conserved structural organization: N-terminally the SLC26/sulphate permease transmembrane domain and C-terminally the cytoplasmic sulphate transporter and anti-sigma factor antagonist (STAS) domain (Geertsma et al., 2015). The STAS domain plays a role in membrane targeting, interaction with scaffolding proteins and other ion channels (detailed later). Furthermore, structural variants in the STAS domain were reported to associate with diseases linked to SLC26 transporters (Sharma et al., 2011). SLC26A9 also has a type-I PDZ-binding domain at the C-terminus (T-A-L), which may promote vesicular trafficking or assembly of macromolecular complexes as seen in other SLC26 members (Lohi et al., 2003; Rossmann et al., 2005). The human SLC26A9 transcript is alternatively spliced, such that inclusion of an exon results in a 96 amino acid extension at the C-terminus (isoform 2). The shorter splice form of 791 residues was designated as canonical isoform, which was also mainly used in heterologous expression systems; however, the isoform-ratio in specific tissues has not yet been elucidated. Similarly to the glycosylation pattern of CFTR, SLC26A9 also displays both core- and complex N-glycosylated forms, which may be important for plasma membrane targeting or function (Li J. et al., 2014; Salomon et al., 2016; Thomson et al., 2016; Bertrand et al., 2017).

The function of SLC26A9 has been widely investigated in heterologous expression systems. Whole-cell patch clamp studies showed that SLC26A9 is a constitutively active chloride channel displaying linear current–voltage characteristics (Dorwart et al., 2007; Loriol et al., 2008; Bertrand et al., 2009; Avella et al., 2011b; Salomon et al., 2016), while the current amplitude was not affected by cAMP stimulation or increase in intracellular Ca^2+^ (Bertrand et al., 2009; Salomon et al., 2016). Interestingly, SLC26A9-expressing epithelial monolayers studied in Ussing chambers displayed constitutive and forskolin stimulated chloride currents, which may be attributed to the enhanced transepithelial electrochemical gradient generated by activation of basolateral potassium channels by cAMP/PKA signaling (Mall et al., 2000; Salomon et al., 2016). Moreover, studies in primary human bronchial epithelial (HBE) cells elegantly demonstrated the presence of a SLC26A9-mediated transepithelial chloride conductance, which contributed to basal and to cAMP/PKA stimulated chloride currents (Bertrand et al., 2009, 2017). These finding support that SLC26A9 could provide an alternative pathway for chloride secretion that may compensate for CFTR dysfunction in CF epithelia. In addition, several studies suggested that SLC26A9 also functions as a chloride-bicarbonate exchanger (Xu et al., 2005; Chang et al., 2009b; Demitrack et al., 2010) and sodium transporter (Chang et al., 2009b). However, the contribution of SLC26A9 to epithelial bicarbonate and sodium transport is not completely understood (see discussion at stomach) and it is possible that transport modes depend on the cell and tissue context.

In the absence of sensitive and specific antibodies for immunolocalization studies, investigations of SLC26A9 largely relied on pharmacological tools. Multiple compounds were found to inhibit SLC26A9 at various potency and selectivity. Highly effective inhibitors include several non-selective chloride channel blockers, such as flufenamic acid, niflumic acid, GlyH-101, and 5-Nitro-2-(3-phenylpropylamino)benzoic acid (NPPB), while anion-exchanger inhibitor 4,4′-Diisothiocyanatostilbene-2,2′-disulfonic acid (DIDS) only partially inhibited SLC26A9 (Dorwart et al., 2007; Loriol et al., 2008; Salomon et al., 2016). On the other hand, SLC26A9 is not sensitive to diphenylamine-2-carboxylic acid (DPC), glibenclamide or CFTRinh172, which makes differentiation from CFTR-mediated chloride currents possible (Loriol et al., 2008; Bertrand et al., 2009; Salomon et al., 2016). Despite the lack of highly selective inhibitors, this characteristic “pharmacological fingerprint” enabled investigation of SLC26A9-mediated chloride secretion in primary epithelial cultures and native tissues (Anagnostopoulou et al., 2012; Bertrand et al., 2017).

The constitutive channel activity suggests that SLC26A9-mediated chloride secretion is controlled by the insertion and stability of SLC26A9 channels in the plasma membrane. Previous studies demonstrated that SLC26A9 cell surface expression is influenced by WNK (with no lysine [K]) kinases that have been implicated in the regulation of cell volume homeostasis and ion transport processes in epithelial cells (Dorwart et al., 2007; Salomon et al., 2016). WNK kinases uniquely function as intracellular chloride and/or osmolality sensors that activate downstream kinases serine/threonine-protein kinase 39 (SPAK) and oxidative stress-responsive 1 protein (OSR1), which can directly modulate ion transporters by phosphorylation (Alessi et al., 2014; Bazúa-Valenti et al., 2015). Furthermore, WNKs can also act as scaffolds that recruit other proteins that regulate the activity or plasma membrane abundance of ion channels (Rodan and Jenny, 2017). This kinase-independent function of WNKs has been demonstrated to affect plasma membrane trafficking of SLC26A9, as well as CFTR, by mechanisms that may involve lysosomal targeting or endosomal sorting (Dorwart et al., 2007; He et al., 2007; Yang et al., 2007; Zhou et al., 2010).

Notably, there is a large overlap in the expression pattern of SLC26A9 and CFTR in epithelial tissues and emerging evidence suggests a complex, multifaceted interaction between the two channels, both under physiological and pathophysiological conditions. First, there is a functional interaction between the two channels mediated by the STAS domain of SLC26A9 and the R domain of CFTR. Although an inhibitory relationship has also been suggested (Chang et al., 2009a), the nature of this molecular interaction is likely stimulatory, as SLC26A9 contributes to cAMP-stimulated chloride currents in HBE cells and co-expression of CFTR with SLC26A9 results in larger chloride currents than expression of CFTR alone (Loriol et al., 2008; Avella et al., 2011b). Interestingly, the cellular background and level of differentiation may be important determinants of this functional interaction between SLC26A9 and CFTR, as suggested by a study that showed a different behavior in human embryonic kidney (HEK293) cells compared to polarized airway epithelial cells (Ousingsawat et al., 2012). Of note, opposite functional interactions between CFTR and SLC26A9 were also reported in two heterologous expression studies that both used HEK cells (Bertrand et al., 2009; Ousingsawat et al., 2012). While these conflicting results are more difficult to reconcile, we speculate that the observed differences in CFTR/SLC26A9 interactions may be related to differences in the cellular background of the HEK cell clones used in the different laboratories.

Biochemical and functional studies of two other SLC26 family members, SLC26A6 and SLC26A3, showed that following cAMP-dependent stimulation, the phosphorylated R domain can bind to STAS domain, which leads to mutual activation of both CFTR and SLC26 transporters (Ko et al., 2004). Coding mutations located in the STAS domain of SLC26A9 may disrupt this stimulatory interaction, as demonstrated by functional analysis of variant L683P (Avella et al., 2011a). Interestingly, STAS domain variant V622L and domain adjacent variant V744M decreased chloride currents of SLC26A9, which could be partially attributed to decreased plasma membrane expression (Chen et al., 2012). Although no disease-association has been reported for the variants above, these studies demonstrate the role of SLC26A9 STAS domain in protein expression, channel function, and stimulation of CFTR currents.

Second, a recent study demonstrated a physical interaction between SLC26A9 and CFTR, which is mediated by PDZ proteins that facilitate trafficking and stabilization of both proteins at the cell surface (Bertrand et al., 2017). As shown by co-localization studies, this interaction is not restricted to the plasma membrane suggesting a common regulatory mechanism along the secretory pathway (Avella et al., 2011b; Bertrand et al., 2017). SLC26A9 shows affinity to a number of PDZ proteins, such as Na^+^/H^+^ exchanger-3 regulatory factor 1 (NHERF1) and CFTR-associated ligand (CAL), which display antagonistic effects in CFTR trafficking (Bertrand et al., 2017). CAL, also known as GOPC (Golgi-associated PDZ and coiled-coil motif-containing protein) facilitates ubiquitination and lysosomal degradation of CFTR, as well as endoplasmic reticulum (ER)-associated degradation of F508del-CFTR, whereas NHERF1 tethers CFTR to the plasma membrane thus promoting its cell surface expression (Cheng and Guggino, 2013; Bergbower et al., 2018). Moreover, F508del-CFTR could be rescued by NHERF1 overexpression or CAL silencing (Wolde et al., 2007; Castellani et al., 2012). Constitutive SLC26A9-mediated chloride secretion is diminished in human bronchial epithelium from CF donors carrying F508del-CFTR and recent evidence suggests a PDZ-domain sensitive, CAL-dependent underlying mechanism (Bertrand et al., 2009, 2017). Due to proteasomal degradation of F508del-CFTR, association of CAL with SLC26A9 increases, which in turn hampers its forward trafficking. Furthermore, restoration of F508del-CFTR by the small molecule CFTR corrector compound VX-809 increased CFTR as well as SLC26A9 cell surface expression indicating that rescue of intracellular processing of CFTR has a positive influence on SLC26A9 membrane targeting. Consistent with this notion, SLC26A9-mediated chloride currents were not affected by co-expression with the CFTR gating mutation G551D, which is inserted into the plasma membrane, but has impaired CFTR chloride channel regulation (Bertrand et al., 2017).

**FIGURE 1:**
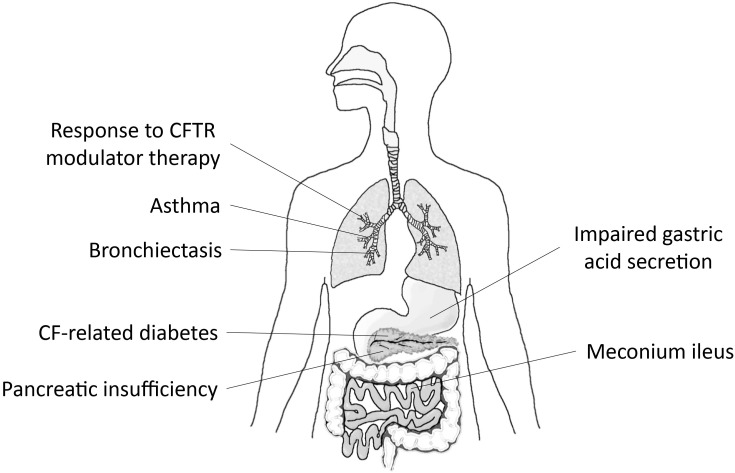
SLC26A9 is a modifier of disease severity in multiple organs affected by CF. Graphical summary depicting associations of variants in *SLC26A9* with exocrine pancreatic insufficiency, CF-related diabetes mellitus (CFRD) and meconium ileus, and emerging data supporting a role of SLC26A9 as modifier of response to CFTR modulator therapy in patients with CF. Beyond CF, SLC26A9 has been implicated in the pathogenesis of asthma, diffuse bronchiectasis (DB), and impaired gastric acid secretion.

Third, numerous genetic association studies and investigations utilizing genetically modified mouse models supported an important role of SLC26A9 in the pathophysiology of several organ manifestations in CF, as well as other chronic lung diseases such as asthma and non-CF bronchiectasis (Anagnostopoulou et al., 2012; Sun et al., 2012; Bakouh et al., 2013; Blackman et al., 2013; Miller et al., 2015; Strug et al., 2016). The results of these studies are consistent with an important role of SLC26A9 as a disease modifier in CF and potentially other muco-obstructive lung diseases and are discussed in more detail below (**Figure 1**).

## SLC26A9 Chloride Channel as a Disease Modifier in CF

### Lung

Muco-obstructive lung disease remains the major cause of morbidity and mortality in patients with CF. The basic CF ion transport defect causes airway surface dehydration, impaired mucociliary clearance and mucus obstruction, which triggers chronic inflammation and infection, and ultimately leads to progressive lung damage (Button et al., 2016). There is a large heterogeneity in lung disease severity among CF patients, which is influenced by genetic and environmental factors. It is estimated that ∼50% of the genetically determined variability can be attributed to the CFTR genotype and the other ∼50% to CF modifier genes (Cutting, 2015). In an effort to identify genetic determinants of CF lung disease, genome-wide association studies (GWAS) were performed in large CF patient cohorts (Wright et al., 2011; Corvol et al., 2015). These studies revealed a number of loci and candidate genes that may influence lung function, but initially, these investigations did not find association with SLC26A9. However, more recently, SLC26A9 was reported as a modifier of lung function and response to CFTR modulator therapy in patients carrying the CFTR gating mutation G551D (Strug et al., 2016). In a cohort of over 1,700 F508del homozygous CF patients and 70 patients with at least one G551D allele the authors examined the effect of SLC26A9 SNP rs7512462, which was previously reported to associate with CF related diabetes, pancreatic insufficiency and meconium ileus. The minor C allele of rs7512462 positively influenced lung function in G551D CF patients, but had no effect in F508del homozygotes. Furthermore, improvement of lung function, as determined from FEV1 response to therapy with the small molecule CFTR potentiator ivacaftor (VX-770) was markedly higher in patients carrying at least one protective C allele. This study also assessed the effect of rs7512462 in primary HBE cultures from F508del homozygous CF patients and found that rescue of cAMP-stimulated currents in cultures pretreated with lumacaftor (VX-809) increased with each additional C allele (Strug et al., 2016). In the presence of an *in vitro* modulatory effect, the lack of SLC26A9 association with lung function in F508del homozygous patients is difficult to reconcile. One explanation may entail the F508del dependent trafficking defect of SLC26A9 and the necessity of CFTR plasma membrane targeting for SLC26A9 function discussed above (Bertrand et al., 2017). On the other hand, age-dependent expression may also be involved. Interestingly, a recent study in a Brazilian cohort of 188 CF patients revealed association of rs7512462 with several outcome measures of pulmonary function (Pereira et al., 2017). Taken together, these data support that SLC26A9 is a modulator of response to CFTR targeted therapies, which in the future may help predict patient outcomes and optimize tailored treatment regimens for individual patients with CF.

Idiopathic diffuse bronchiectasis (DB) is a chronic lung disease involving airway mucus obstruction, recurrent infections, airway inflammation, and remodeling that resembles CF lung disease in many aspects (Bergougnoux et al., 2015). Interestingly, a previous study reported rare loss-of-function SLC26A9 mutations in patients with DB suggesting that SLC26A9 may be implicated in disease pathogenesis. In a study comparing 147 patients with DB, 78 patients with CF and 50 healthy controls, exon sequencing of SLC26A9 revealed coding variants R575Y and V486I in two DB patients (Bakouh et al., 2013). Functional analysis showed that both mutations abolished chloride currents and that R575Y located in the STAS domain also interfered with the stimulatory interaction with CFTR.

Further insights on the importance of SLC26A9 in airway epithelial chloride secretion, airway surface hydration, and mucus clearance came from studies in mice with allergic airway disease. In a murine model of allergic asthma, it was shown that type 2 airway inflammation induced SLC26A9-mediated Cl^−^ secretion, which prevented airway mucus plugging in presence of mucus hypersecretion (Anagnostopoulou et al., 2010, 2012). In the absence of allergic airway inflammation, *Slc26a9*-deficient (*Slc26a9*^−/−^) mice displayed normal lung morphology and no differences in epithelial ion transport when compared to wild-type mice. However, in the presence of allergic inflammation *Slc26a9*^−/−^ mice lacked upregulation of chloride secretion and mucus hypersecretion led to airway mucus plugging. Furthermore, three SNPs at 3′ UTR of SLC26A9 were identified that were associated with a risk for childhood asthma, with an odds ratio of 1.48. Functional investigations revealed that one of these non-coding variants altered a putative micro-RNA response element in the 3′ UTR of SLC26A9 and lead to micro-RNA mediated translational repression *in vitro*, indicating that decreased SLC26A9 protein expression may be the underlying mechanism for disease association (Anagnostopoulou et al., 2012).

### Stomach

Investigations in the stomach revealed an essential role of SLC26A9 in the regulation of gastric acid secretion. In the gastric mucosa SLC26A9 is expressed in the surface epithelial cells and in the parietal cells of the deep gastric gland. *Slc26a9*^−/−^ mice displayed structural and ultrastructural changes, such as distended gastric glands, hypoplasia of parietal cells, and loss of tubulovesicular system inside the parietal cells (Xu et al., 2008). Furthermore, H-K-ATPase expression was decreased. *Slc26a9^−/−^* mice had complete achlorhydria, which could be explained by the absence of transmucosal chloride secretion and/or the role of Slc26a9 in the maintenance and maturation of the secretory pathway that regulates the H-K-ATPase trafficking. Alternatively, Slc26a9 could also promote gastric bicarbonate secretion and regulation of luminal pH to protect against injury by gastric acid (Xu et al., 2005; Demitrack et al., 2010). It is currently debated how SLC26A9 could contribute to bicarbonate secretion. One possibility would be that SLC26A9-mediated chloride secretion is paralleled by chloride-bicarbonate exchange via apical anion exchangers, similarly to the proposed model of pancreatic fluid secretion, where CFTR works tandem with anion exchangers to secrete bicarbonate (Stewart et al., 2009). The second possibility is that SLC26A9 itself can operate as an anion exchanger, which was supported by two studies on gastric epithelium (Xu et al., 2005; Demitrack et al., 2010), however, most heterologous expression data suggest that SLC26A9 functions as an ion channel with minimal bicarbonate conductance. Third, the channel conductance to bicarbonate could also be modulated, similarly to the regulation of CFTR bicarbonate permeability by WNK1-OSR1/SPAK pathway (Park et al., 2010). Notably, SLC26A9 expression was upregulated in *Helicobacter pylori* infection, as well as upon chronic gastritis induced by interleukin-11 in mice, indicating that SLC26A9 is a key player in gastric mucosal defense under pathological conditions (Henriksnäs et al., 2006; Howlett et al., 2012).

### Intestine

Obstruction of the ileum due to highly viscous and sticky bowel content, also known as meconium ileus, affects ∼15% of newborns with CF. Sibling and twin studies demonstrated a high heritability for this disease phenotype and a number of genetic studies reported association of SLC26A9 with meconium ileus (Blackman et al., 2006; Sun et al., 2012; Li W. et al., 2014; Miller et al., 2015). SLC26A9 expression decreases along the GI tract with relatively high levels in the stomach, medium levels in the proximal duodenum, low levels in the ileum and no detectable expression in the colon, whereas CFTR shows an inverse pattern with low expression in the stomach and high levels in the duodenum, small intestine and colon (Liu et al., 2015). *Cftr*-deficient (*Cftr^−/−^*) mice show a severe meconium ileus-like intestinal phenotype characterized by severe intestinal plugging. When *Cftr^−/−^* mice were crossbred with *Slc26a9^−/−^* mice, mortality highly increased, indicating that SLC26A9-mediated anion secretion ameliorates meconium ileus (Liu et al., 2015). *Slc26a9^−/−^* mice also failed to elicit duodenal bicarbonate secretion in response to acid load, which may signify the physiological role of SLC26A9 to neutralize gastric acid in the duodenum (Singh et al., 2013).

### Pancreas

Exocrine pancreatic insufficiency (PI) in CF is present from early life in nearly all patients with severe CFTR genotypes. CFTR is expressed in the pancreatic ductal epithelium and regulates the secretion of an alkaline, bicarbonate rich pancreatic fluid that washes out digestive enzymes produced by acinar cells. CFTR dysfunction leads to an acidic, low volume, protein rich secretion that can slow down or even block the outflow of zymogens from the ductal tree (Kopelman et al., 1988). Early histopathological changes include dilatation of ducts filled with inspissated zymogen material and destruction of acinar cells (Tucker et al., 2003). In advanced stages, fibrotic and adipose tissue replaces acinar mass and destroys Langerhans-islands (Gibson-Corley et al., 2016). PI shows a strong, but incomplete correlation with CF genotype and CFTR chloride channel function, while SLC26A9 can largely explain the remainder of genetic variability (Cystic Fibrosis Genotype-Phenotype Consortium, 1993; Hirtz et al., 2004; Li W. et al., 2014; Miller et al., 2015). These studies found correlation between circulating levels of immunoreactive trypsinogen (IRT), an early biomarker for exocrine damage, and SLC26A9 SNPs that also associated with meconium ileus and CF-related diabetes mellitus (CFRD). The physiological function of SLC26A9 in the pancreas is largely unexplored. Emerging data suggest that SLC26A9 may enhance ductal anion transport and fluid secretion (Li et al., 2016). Impaired SLC26A9-mediated secretion may thus aggravate the defective ductal wash-out mechanism and ductal plugging in CF. Reduced fluid secretion and ductal mucus obstruction are also present in chronic pancreatitis, a disease that shares many key pathogenic features with CF of the pancreas (Maléth et al., 2015; Balázs et al., 2018). It is conceivable that SLC26A9 may also act as a modifier in these disorders.

Pancreatic insufficiency confers a major risk for CFRD. CFRD is an age-dependent complication that affects 2% of children and 50% of adults with high heritability (Blackman et al., 2009; Moran et al., 2009). A GWAS identified major association of SLC26A9 variants with CFRD, with a hazards ratio of 1.47. Interestingly, meta-analysis of two GWAS studies in type II diabetes showed evidence for association with the same SLC26A9 SNPs, although the risk allele was different (Blackman et al., 2013). There are two prevailing hypotheses on CFRD development: one is that CFRD is driven by the exocrine damage that stresses endocrine cells and causes islet remodeling (Rotti et al., 2018). This is supported by a Mendelian randomization study utilizing a disease-associated SLC26A9 SNP, where a causal relationship between PI and CFRD risk was determined (Soave et al., 2014). Alternatively, loss of CFTR function might have a direct role in insulin secretion (Guo et al., 2014) and/or result in an intrinsic defect that impairs endocrine function before acinar damage occurs (Gibson-Corley et al., 2016). Given its function as chloride channel, it is possible that SLC26A9 may regulate membrane potential and insulin secretion in β-cells, however, there are no data available that support expression of SLC26A9 in the endocrine pancreas.

## Conclusion and Future Directions

**FIGURE 2:**
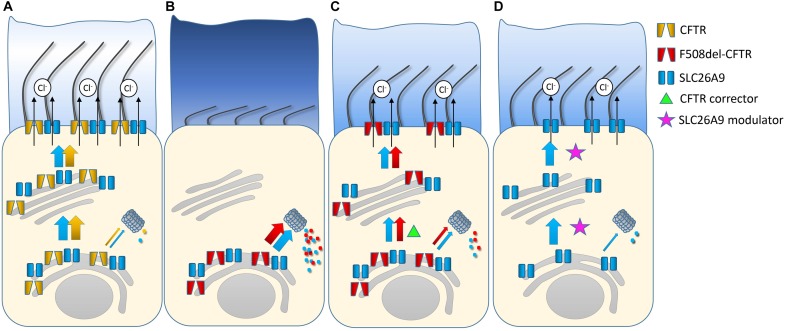
Role SLC26A9 in healthy airways and as a therapeutic target in cystic fibrosis (CF). Healthy airway epithelia express SLC26A9 and CFTR that are co-trafficked from the ER to the plasma membrane, where SLC26A9 functions as a constitutive and CFTR as a cAMP/PKA-regulated chloride channel. Further, SLC26A9 and CFTR interact reciprocally to augment transepithelial chloride/fluid secretion essential to maintain proper airway surface hydration and effective mucociliary clearance **(A)**. In airway epithelial cells from patients with CF carrying the most common disease-causing mutation F508del-CFTR, impaired folding of F508del leads to its retention in the ER and degradation by the proteasome. The F508del trafficking defect also hampers co-trafficking of SLC26A9 and its insertion into the plasma membrane. Lack of SLC26A9 chloride channels may aggravate deficient chloride secretion in CF **(B)**. Pharmacological rescue of F508del-CFTR trafficking with the CFTR corrector lumacaftor (VX-809) restores co-trafficking and insertion of SLC26A9 chloride channels into the apical plasma membrane **(C)**. Compounds that facilitate trafficking of SLC26A9 in the absence of CFTR may improve chloride secretion and airway surface hydration in all patients with CF regardless of the type of CFTR mutation. Alternatively, chloride secretion may also be facilitated by compounds that potentiate SLC26A9 channels that are present and/or newly trafficked to the plasma membrane **(D)**.

In summary, SLC26A9 constitutes an alternative chloride channel that is implicated in coordinated ion and fluid secretion in various epithelial tissues affected in CF including the airways, the pancreas and the gastro-intestinal tract (Lohi et al., 2002; Dorwart et al., 2007; Bertrand et al., 2009). Demonstration of its role as a disease modifier, as well as overlapping expression of SLC26A9 and CFTR in several affected organs, suggest SLC26A9 as an attractive alternative therapeutic target to bypass the primary ion transport defect in CF (Sun et al., 2012; Blackman et al., 2013; Miller et al., 2015; Strug et al., 2016). In the airways, activation of SLC26A9-mediated chloride/fluid secretion is predicted to counteract airway surface dehydration that sets the stage for mucus plugging and mucociliary dysfunction and constitutes an important disease mechanism in CF lung disease (Mall and Hartl, 2014). Similarly, in the gastro-intestinal tract, activation of SLC26A9 chloride channels is expected to improve the hydration of intestinal surfaces and facilitate pancreatic fluid secretion thus counteracting important extra-pulmonary organ dysfunctions in CF. Therefore, SLC26A9 is an attractive target not only for the treatment of CF lung disease, but also for systemic therapy that may be beneficial for the prevention and/or treatment of several extrapulmonary disease manifestations including meconium ileus and distal intestinal obstruction syndrome (DIOS), exocrine pancreatic insufficiency and CFRD. Further, pharmacological activation of SLC26A9-mediated chloride/fluid secretion may also be beneficial in other muco-obstructive lung diseases and in chronic pancreatitis (Mall, 2016; Balázs et al., 2018). Importantly activation of an alternative chloride channel is expected to be beneficial for all patients with CF, irrespective of their *CFTR* genotype, including patients with non-sense or splicing mutations, where no CFTR protein is made, or other rare CFTR mutations that do not respond to emerging CFTR modulator therapies (Li et al., 2017). Given the reciprocal interaction with CFTR (**Figures 2A–C**), pharmacological activation of SLC26A9 may also augment functional rescue of mutant CFTR achieved by CFTR modulator therapies (Strug et al., 2016; Bertrand et al., 2017). However, to date, no compounds that activate SLC26A9 chloride channel function have been reported and the development of such SLC26A9 modulators remains a major challenge for further testing of these concepts in preclinical models and in the clinical arena. In this context, the negative impact of the common CFTR mutation F508del present on at least one allele in ∼90% of CF patients on SLC26A9 trafficking indicates that an ideal SLC26A9 modulator compound should overcome this interaction and facilitate trafficking of SLC26A9 from the ER to the plasma membrane independent of CFTR co-trafficking (Strug et al., 2016; Bertrand et al., 2017; **Figure 2D**). In addition to compounds that facilitate SLC26A9 trafficking to increase the number of channels, SLC26A9-mediated chloride secretion may be augmented by compounds that enhance the open probability of the channel or its stability in the plasma membrane. Single channel recordings demonstrated SLC26A9 channels with open and closed states and a conductance of ∼3.2 pS (Chang et al., 2009b) suggesting that it may be possible to increase the open probability by SLC26A9 potentiator compounds. Taking the development of CFTR modulators as a model for drug development, such SCL26A9 modulators may be identified by high-throughput screening of compound libraries in SLC26A9-expression epithelial cells (Gentzsch and Mall, 2018). In addition, a better understanding of SLC26A9 regulation in epithelial tissues, including its interaction network and signaling pathways, may help to explore SLC26A9 as a novel therapeutic target in CF and potentially other muco-obstructive lung diseases.

## Author Contributions

AB and MM conceived and designed the review, contributed to analysis and interpretation of published literature, and drafted the article and revised it critically for important intellectual content.

## Conflict of Interest Statement

The authors declare that the research was conducted in the absence of any commercial or financial relationships that could be construed as a potential conflict of interest.

## References

[B1] Alessi D. R. Zhang J. Khanna A. Hochdörfer T. Shang Y. Kahle K. T. (2014). The WNK-SPAK/OSR1 pathway: master regulator of cation-chloride cotransporters. *Sci. Signal.* 7:re3. 10.1126/scisignal.2005365 25028718

[B2] Anagnostopoulou P. Dai L. Schatterny J. Hirtz S. Duerr J. Mall M. A. (2010). Allergic airway inflammation induces a pro-secretory epithelial ion transport phenotype in mice. *Eur. Respir. J.* 36 1436–1447. 10.1183/09031936.00181209 20413543

[B3] Anagnostopoulou P. Riederer B. Duerr J. Michel S. Binia A. Agrawal R. (2012). SLC26A9-mediated chloride secretion prevents mucus obstruction in airway inflammation. *J. Clin. Invest.* 122 3629–3634. 10.1172/JCI60429 22945630 PMC3461899

[B4] Avella M. Borgese F. Ehrenfeld J. (2011a). Characterization of the L683P mutation of SLC26A9 in Xenopus oocytes. *Biochim. Biophys. Acta* 1810 577–583. 10.1016/j.bbagen.2011.03.012 21439353

[B5] Avella M. Loriol C. Boulukos K. Borgese F. Ehrenfeld J. (2011b). SLC26A9 stimulates CFTR expression and function in human bronchial cell lines. *J. Cell. Physiol.* 226 212–223. 10.1002/jcp.22328 20658517

[B6] Bakouh N. Bienvenu T. Thomas A. Ehrenfeld J. Liote H. Roussel D. (2013). Characterization of SLC26A9 in patients with CF-like lung disease. *Hum. Mutat.* 34 1404–1414. 10.1002/humu.22382 24272871

[B7] Balázs A. Balla Z. Kui B. Maléth J. Rakonczay Z. Duerr J. (2018). Ductal mucus obstruction and reduced fluid secretion are early defects in chronic pancreatitis. *Front. Physiol.* 9:632. 10.3389/fphys.2018.00632 29896115 PMC5987707

[B8] Bazúa-Valenti S. Chávez-Canales M. Rojas-Vega L. González-Rodríguez X. Vázquez N. Rodríguez-Gama A. (2015). The effect of WNK4 on the Na+–Cl- cotransporter is modulated by intracellular chloride. *J. Am. Soc. Nephrol.* 26 1781–1786. 10.1681/ASN.2014050470 25542968 PMC4520168

[B9] Bergbower E. Boinot C. Sabirzhanova I. Guggino W. Cebotaru L. (2018). The CFTR-associated ligand arrests the trafficking of the mutant ΔF508 CFTR channel in the ER contributing to cystic fibrosis. *Cell. Physiol. Biochem.* 45 639–655. 10.1159/000487120 29402832 PMC5861351

[B10] Bergougnoux A. Viart V. Miro J. Bommart S. Molinari N. des Georges M. (2015). Should diffuse bronchiectasis still be considered a CFTR-related disorder? *J. Cyst. Fibros.* 14 646–653. 10.1016/j.jcf.2015.02.012 25797027

[B11] Bertrand C. A. Mitra S. Mishra S. K. Wang X. Zhao Y. Pilewski J. M. (2017). The CFTR trafficking mutation F508del inhibits the constitutive activity of SLC26A9. *Am. J. Physiol. Lung. Cell Mol. Physiol.* 312 L912–L925. 10.1152/ajplung.00178.2016 28360110 PMC5495941

[B12] Bertrand C. A. Zhang R. Pilewski J. M. Frizzell R. A. (2009). SLC26A9 is a constitutively active, CFTR-regulated anion conductance in human bronchial epithelia. *J. Gen. Physiol.* 133 421–438. 10.1085/jgp.200810097 19289574 PMC2664976

[B13] Blackman S. M. Commander C. W. Watson C. Arcara K. M. Strug L. J. Stonebraker J. R. (2013). Genetic modifiers of cystic fibrosis-related diabetes. *Diabetes Metab. Res. Rev.* 62 3627–3635. 10.2337/db13-0510 23670970 PMC3781476

[B14] Blackman S. M. Deering-Brose R. McWilliams R. Naughton K. Coleman B. Lai T. (2006). Relative contribution of genetic and nongenetic modifiers to intestinal obstruction in cystic fibrosis. *Gastroenterology* 131 1030–1039. 10.1053/j.gastro.2006.07.016 17030173 PMC1764617

[B15] Blackman S. M. Hsu S. Vanscoy L. L. Collaco J. M. Ritter S. E. Naughton K. (2009). Genetic modifiers play a substantial role in diabetes complicating cystic fibrosis. *J. Clin. Endocrinol. Metab.* 94 1302–1309. 10.1210/jc.2008-2186 19126627 PMC2682466

[B16] Button B. Anderson W. H. Boucher R. C. (2016). Mucus hyperconcentration as a unifying aspect of the chronic bronchitic phenotype. *Ann. Am. Thorac. Soc.* 13(Suppl. 2), S156–S162. 10.1513/AnnalsATS.201507-455KV 27115951 PMC5015722

[B17] Castellani S. Guerra L. Favia M. Di Gioia S. Casavola V. Conese M. (2012). NHERF1 and CFTR restore tight junction organisation and function in cystic fibrosis airway epithelial cells: role of ezrin and the RhoA/ROCK pathway. *Lab. Invest.* 92 1527–1540. 10.1038/labinvest.2012.123 22964850

[B18] Chang M.-H. Plata C. Sindic A. Ranatunga W. K. Chen A.-P. Zandi-Nejad K. (2009a). Slc26a9 is inhibited by the R-region of the cystic fibrosis transmembrane conductance regulator via the STAS domain. *J. Biol. Chem.* 284 28306–28318. 10.1074/jbc.M109.001669 19643730 PMC2788881

[B19] Chang M.-H. Plata C. Zandi-Nejad K. Sinđić A. Sussman C. R. Mercado A. (2009b). Slc26A9 - anion exchanger, channel and Na+ transporter. *J. Membr. Biol.* 228 125–140. 10.1007/s00232-009-9165-5 19365592 PMC2733867

[B20] Chen A.-P. Chang M.-H. Romero M. F. (2012). Functional analysis of non-synonymous single nucleotide polymorphisms in human SLC26A9. *Hum. Mutat.* 33 1275–1284. 10.1002/humu.22107 22544634 PMC3399991

[B21] Cheng J. Guggino W. (2013). Ubiquitination and degradation of CFTR by the E3 ubiquitin ligase MARCH2 through its association with adaptor proteins CAL and STX6. *PLoS One* 8:e68001. 10.1371/journal.pone.0068001 23818989 PMC3688601

[B22] Consortium G. (2017). Genetic effects on gene expression across human tissues. *Nature* 550 204–213. 10.1038/nature24277 29022597 PMC5776756

[B23] Corvol H. Blackman S. M. Boëlle P.-Y. Gallins P. J. Pace R. G. Stonebraker J. R. (2015). Genome-wide association meta-analysis identifies five modifier loci of lung disease severity in cystic fibrosis. *Nat. Commun.* 6:8382. 10.1038/ncomms9382 26417704 PMC4589222

[B24] Cutting G. R. (2015). Cystic fibrosis genetics: from molecular understanding to clinical application. *Nat. Rev. Genet.* 16 45–56. 10.1038/nrg3849 25404111 PMC4364438

[B25] Cystic Fibrosis Genotype-Phenotype Consortium (1993). Correlation between genotype and phenotype in patients with cystic fibrosis. *N. Engl. J. Med.* 329 1308–1313. 10.1056/NEJM199310283291804 8166795

[B26] Demitrack E. S. Soleimani M. Montrose M. H. (2010). Damage to the gastric epithelium activates cellular bicarbonate secretion via SLC26A9 Cl-/HCO3- exchange. *Am. J. Physiol. Gastrointest. Liver Physiol.* 299 G255–G264. 10.1152/ajpgi.00037.2010 20413716 PMC2904109

[B27] Dorwart M. R. Shcheynikov N. Wang Y. Stippec S. Muallem S. (2007). SLC26A9 is a Cl(-) channel regulated by the WNK kinases. *J. Physiol.* 584 333–345. 10.1113/jphysiol.2007.135855 17673510 PMC2277069

[B28] Geertsma E. R. Chang Y.-N. Shaik F. R. Neldner Y. Pardon E. Steyaert J. (2015). Structure of a prokaryotic fumarate transporter reveals the architecture of the SLC26 family. *Nat. Struct. Mol. Biol.* 22 803–808. 10.1038/nsmb.3091 26367249

[B29] Gentzsch M. Mall M. A. (2018). Ion channel modulators in cystic fibrosis. *Chest* 154 383–393. 10.1016/j.chest.2018.04.036 29750923 PMC6113631

[B30] Gibson-Corley K. N. Meyerholz D. K. Engelhardt J. F. (2016). Pancreatic pathophysiology in cystic fibrosis. *J. Pathol.* 238 311–320. 10.1002/path.4634 26365583 PMC4699289

[B31] Guo J. H. Chen H. Ruan Y. C. Zhang X. L. Zhang X. H. Fok K. L. (2014). Glucose-induced electrical activities and insulin secretion in pancreatic islet β-cells are modulated by CFTR. *Nat. Commun.* 5:4420. 10.1038/ncomms5420 25025956 PMC4104438

[B32] He G. Wang H.-R. Huang S.-K. Huang C.-L. (2007). Intersectin links WNK kinases to endocytosis of ROMK1. *J. Clin. Invest.* 117 1078–1087. 10.1172/JCI30087 17380208 PMC1821066

[B33] Henriksnäs J. Phillipson M. Storm M. Engstrand L. Soleimani M. Holm L. (2006). Impaired mucus-bicarbonate barrier in Helicobacter pylori-infected mice. *Am. J. Physiol. Gastrointest. Liver Physiol.* 291 G396–G403. 10.1152/ajpgi.00017.2006 16614375

[B34] Hirtz S. Gonska T. Seydewitz H. H. Thomas J. Greiner P. Kuehr J. (2004). CFTR Cl- channel function in native human colon correlates with the genotype and phenotype in cystic fibrosis. *Gastroenterology* 127 1085–1095. 10.1053/j.gastro.2004.07.006 15480987

[B35] Howlett M. Chalinor H. V. Buzzelli J. N. Nguyen N. van Driel I. R. Bell K. M. (2012). IL-11 is a parietal cell cytokine that induces atrophic gastritis. *Gut* 61 1398–1409. 10.1136/gutjnl-2011-300539 22180059 PMC3471558

[B36] Ko S. B. H. Zeng W. Dorwart M. R. Luo X. Kim K. H. Millen L. (2004). Gating of CFTR by the STAS domain of SLC26 transporters. *Nat. Cell Biol.* 6 343–350. 10.1038/ncb1115 15048129 PMC3943213

[B37] Kopelman H. Corey M. Gaskin K. Durie P. Weizman Z. Forstner G. (1988). Impaired chloride secretion, as well as bicarbonate secretion, underlies the fluid secretory defect in the cystic fibrosis pancreas. *Gastroenterology* 95 349–355. 10.1016/0016-5085(88)90490-8 3391365

[B38] Lee H. J. Yoo J. E. Namkung W. Cho H.-J. Kim K. Kang J. W. (2015). Thick airway surface liquid volume and weak mucin expression in pendrin-deficient human airway epithelia. *Physiol. Rep.* 3:e12480. 10.14814/phy2.12480 26243215 PMC4562566

[B39] Li H. Salomon J. J. Sheppard D. N. Mall M. A. Galietta L. J. (2017). Bypassing CFTR dysfunction in cystic fibrosis with alternative pathways for anion transport. *Curr. Opin. Pharmacol.* 34 91–97. 10.1016/j.coph.2017.10.002 29065356

[B40] Li J. Xia F. Reithmeier R. A. F. (2014). N-glycosylation and topology of the human SLC26 family of anion transport membrane proteins. *Am. J. Physiol. Cell Physiol.* 306 C943–C960. 10.1152/ajpcell.00030.2014 24647542

[B41] Li T. Riederer B. Liu X. Pallagi P. Singh A. K. Soleimani M. (2016). Tu1491 loss of Slc26a9 anion transporter results in reduced pancreatic fluid secretion in young female mice. *Gastroenterology* 150:S915 10.1016/S0016-5085(16)33103-1

[B42] Li W. Soave D. Miller M. R. Keenan K. Lin F. Gong J. (2014). Unraveling the complex genetic model for cystic fibrosis: pleiotropic effects of modifier genes on early cystic fibrosis-related morbidities. *Hum. Genet.* 133 151–161. 10.1007/s00439-013-1363-7 24057835

[B43] Liu X. Li T. Riederer B. Lenzen H. Ludolph L. Yeruva S. (2015). Loss of Slc26a9 anion transporter alters intestinal electrolyte and HCO3- transport and reduces survival in CFTR-deficient mice. *Pflugers Arch.* 467 1261–1275. 10.1007/s00424-014-1543-x 24965066 PMC4434866

[B44] Lohi H. Kujala M. Makela S. Lehtonen E. Kestila M. Saarialho-Kere U. (2002). Functional characterization of three novel tissue-specific anion exchangers SLC26A7, -A8, and -A9. *J. Biol. Chem.* 277 14246–14254. 10.1074/jbc.M111802200 11834742

[B45] Lohi H. Lamprecht G. Markovich D. Heil A. Kujala M. Seidler U. (2003). Isoforms of SLC26A6 mediate anion transport and have functional PDZ interaction domains. *Am. J. Physiol Cell Physiol.* 284 C769–C779. 10.1152/ajpcell.00270.2002 12444019

[B46] Loriol C. Dulong S. Avella M. Gabillat N. Boulukos K. Borgese F. (2008). Characterization of SLC26A9, facilitation of Cl(-) transport by bicarbonate. *Cell. Physiol. Biochem.* 22 15–30. 10.1159/000149780 18769029

[B47] Maléth J. Balázs A. Pallagi P. Balla Z. Kui B. Katona M. (2015). Alcohol disrupts levels and function of the cystic fibrosis transmembrane conductance regulator to promote development of pancreatitis. *Gastroenterology* 148 427–439.e16. 10.1053/j.gastro.2014.11.002 25447846 PMC4353632

[B48] Mall M. Wissner A. Schreiber R. Kuehr J. Seydewitz H. H. Brandis M. (2000). Role of K(V)LQT1 in cyclic adenosine monophosphate-mediated Cl(-) secretion in human airway epithelia. *Am. J. Respir. Cell Mol. Biol.* 23 283–289. 10.1165/ajrcmb.23.3.4060 10970817

[B49] Mall M. A. (2016). Unplugging mucus in cystic fibrosis and chronic obstructive pulmonary disease. *Ann. Am. Thorac. Soc.* 13(Suppl. 2), S177–S185. 10.1513/AnnalsATS.201509-641KV 27115954

[B50] Mall M. A. Hartl D. (2014). CFTR: cystic fibrosis and beyond. *Eur. Respir. J.* 44 1042–1054. 10.1183/09031936.00228013 24925916

[B51] Miller M. R. Soave D. Li W. Gong J. Pace R. G. Boëlle P.-Y. (2015). Variants in solute carrier SLC26A9 modify prenatal exocrine pancreatic damage in cystic fibrosis. *J. Pediatr.* 166 1152–1157.e6. 10.1016/j.jpeds.2015.01.044 25771386 PMC4530786

[B52] Moran A. Dunitz J. Nathan B. Saeed A. Holme B. Thomas W. (2009). Cystic fibrosis–related diabetes: current trends in prevalence, incidence, and mortality. *Diabetes Care* 32 1626–1631. 10.2337/dc09-0586 19542209 PMC2732133

[B53] Ousingsawat J. Schreiber R. Kunzelmann K. (2012). Differential contribution of SLC26A9 to Cl- conductance in polarized and non-polarized epithelial cells. *J. Cell. Physiol.* 227 2323–2329. 10.1002/jcp.22967 21809345

[B54] Park H. W. Nam J. H. Kim J. Y. Namkung W. Yoon J. S. Lee J.-S. (2010). Dynamic regulation of CFTR bicarbonate permeability by [Cl-]i and its role in pancreatic bicarbonate secretion. *Gastroenterology* 139 620–631. 10.1053/j.gastro.2010.04.004 20398666

[B55] Pereira S. V.-N. Ribeiro J. D. Bertuzzo C. S. Marson F. A. L. (2017). Association of clinical severity of cystic fibrosis with variants in the SLC gene family (SLC6A14, SLC26A9, SLC11A1 and SLC9A3). *Gene* 629 117–126. 10.1016/j.gene.2017.07.068 28756021

[B56] Ratjen F. Hug C. Marigowda G. Tian S. Huang X. Stanojevic S. (2017). Efficacy and safety of lumacaftor and ivacaftor in patients aged 6-11 years with cystic fibrosis homozygous for F508del-CFTR: a randomised, placebo-controlled phase 3 trial. *Lancet Respir. Med.* 5 557–567. 10.1016/S2213-2600(17)30215-1 28606620

[B57] Rodan A. R. Jenny A. (2017). WNK kinases in development and disease. *Curr. Top. Dev. Biol* 123 1–47. 10.1016/bs.ctdb.2016.08.004 28236964 PMC5329892

[B58] Rossmann H. Jacob P. Baisch S. Hassoun R. Meier J. Natour D. (2005). The CFTR associated protein CAP70 interacts with the Apical Cl-/HCO3- exchanger DRA in rabbit small intestinal mucosa. *Biochemistry* 44 4477–4487. 10.1021/bi048828b 15766278

[B59] Rotti P. G. Xie W. Poudel A. Yi Y. Sun X. Tyler S. R. (2018). Pancreatic and islet remodeling in cystic fibrosis transmembrane conductance regulator (CFTR) knockout ferrets. *Am. J. Pathol.* 188 876–890. 10.1016/j.ajpath.2017.12.015 29366680 PMC5963477

[B60] Salomon J. J. Spahn S. Wang X. Füllekrug J. Bertrand C. A. Mall M. A. (2016). Generation and functional characterization of epithelial cells with stable expression of SLC26A9 Cl- channels. *Am. J. Physiol. Lung. Cell. Mol. Physiol.* 310 L593–L602. 10.1152/ajplung.00321.2015 26801567 PMC4888553

[B61] Sharma A. K. Rigby A. C. Alper S. L. (2011). STAS domain structure and function. *Cell. Physiol. Biochem.* 28 407–422. 10.1159/000335104 22116355 PMC3709189

[B62] Singh A. K. Liu Y. Riederer B. Engelhardt R. Thakur B. K. Soleimani M. (2013). Molecular transport machinery involved in orchestrating luminal acid-induced duodenal bicarbonate secretion *in vivo*. *J. Physiol.* 591 5377–5391. 10.1113/jphysiol.2013.254854 24018950 PMC3936374

[B63] Soave D. Miller M. R. Keenan K. Li W. Gong J. Ip W. (2014). Evidence for a causal relationship between early exocrine pancreatic disease and cystic fibrosis–related diabetes: a mendelian randomization study. *Diabetes Metab. Res. Rev.* 63 2114–2119. 10.2337/db13-1464 24550193 PMC4030111

[B64] Sosnay P. R. Siklosi K. R. Van Goor F. Kaniecki K. Yu H. Sharma N. (2013). Defining the disease liability of variants in the cystic fibrosis transmembrane conductance regulator gene. *Nat. Genet.* 45 1160–1167. 10.1038/ng.2745 23974870 PMC3874936

[B65] Stewart A. K. Yamamoto A. Nakakuki M. Kondo T. Alper S. L. Ishiguro H. (2009). Functional coupling of apical Cl-/HCO3- exchange with CFTR in stimulated HCO3- secretion by guinea pig interlobular pancreatic duct. *Am. J. Physiol. Gastrointest. Liver Physiol.* 296 G1307–G1317. 10.1152/ajpgi.90697.2008 19342507 PMC2697944

[B66] Strug L. J. Gonska T. He G. Keenan K. Ip W. Boëlle P.-Y. (2016). Cystic fibrosis gene modifier SLC26A9 modulates airway response to CFTR-directed therapeutics. *Hum. Mol. Genet.* 25 4590–4600. 10.1093/hmg/ddw290 28171547 PMC5886039

[B67] Sun L. Rommens J. M. Corvol H. Li W. Li X. Chiang T. A. (2012). Multiple apical plasma membrane constituents are associated with susceptibility to meconium ileus in individuals with cystic fibrosis. *Nat. Genet.* 44 562–569. 10.1038/ng.2221 22466613 PMC3371103

[B68] Thomson R. B. Thomson C. L. Aronson P. S. (2016). N-glycosylation critically regulates function of oxalate transporter SLC26A6. *Am. J. Physiol. Cell Physiol.* 311 C866–C873. 10.1152/ajpcell.00171.2016 27681177 PMC5206297

[B69] Tucker J. A. Spock A. Spicer S. S. Shelburne J. D. Bradford W. (2003). Inspissation of pancreatic zymogen material in cystic fibrosis. *Ultrastruct. Pathol.* 27 323–335. 10.1080/716100784 14708723

[B70] Welsh M. J. Smith A. E. (1993). Molecular mechanisms of CFTR chloride channel dysfunction in cystic fibrosis. *Cell* 73 1251–1254. 10.1016/0092-8674(93)90353-R 7686820

[B71] Wolde M. Fellows A. Cheng J. Kivenson A. Coutermarsh B. Talebian L. (2007). Targeting CAL as a negative regulator of ΔF508-CFTR cell-surface expression an rna interference and structure-based mutagenetic approaCH. *J. Biol. Chem.* 282 8099–8109. 10.1074/jbc.M611049200 17158866

[B72] Wright F. A. Strug L. J. Doshi V. K. Commander C. W. Blackman S. M. Sun L. (2011). Genome-wide association and linkage identify modifier loci of lung disease severity in cystic fibrosis at 11p13 and 20q13.2. *Nat. Genet.* 43 539–546. 10.1038/ng.838 21602797 PMC3296486

[B73] Xu J. Henriksnäs J. Barone S. Witte D. Shull G. E. Forte J. G. (2005). SLC26A9 is expressed in gastric surface epithelial cells, mediates Cl-/HCO3- exchange, and is inhibited by NH4+. *Am. J. Physiol. Cell Physiol.* 289 C493–C505. 10.1152/ajpcell.00030.2005 15800055

[B74] Xu J. Song P. Miller M. L. Borgese F. Barone S. Riederer B. (2008). Deletion of the chloride transporter Slc26a9 causes loss of tubulovesicles in parietal cells and impairs acid secretion in the stomach. *Proc. Natl. Acad. Sci. U.S.A.* 105 17955–17960. 10.1073/pnas.0800616105 19004773 PMC2582584

[B75] Yang C.-L. Liu X. Paliege A. Zhu X. Bachmann S. Dawson D. C. (2007). WNK1 and WNK4 modulate CFTR activity. *Biochem. Biophys. Res. Commun.* 353 535–540. 10.1016/j.bbrc.2006.11.151 17194447

[B76] Zhou B. Zhuang J. Gu D. Wang H. Cebotaru L. Guggino W. B. (2010). WNK4 enhances the degradation of NCC through a sortilin-mediated lysosomal pathway. *J. Am. Soc. Nephrol.* 21 82–92. 10.1681/ASN.2008121275 19875813 PMC2799281

